# Effects of xylanase and phytase supplementation in diets containing *Moringa oleifera* leaf meal on intestinal morphology and the relative size and weight of internal organs of laying hens

**DOI:** 10.3389/fvets.2025.1615214

**Published:** 2025-07-18

**Authors:** Gabriel Miranda Macambira, Carlos Bôa-Viagem Rabello, Hélia Sharlane de Holanda Oliveira, Marcos José Batista dos Santos, Apolônio Gomes Ribeiro, Oziel Saturnino Lins Júnior, Lucas Delano Nascimento de Sousa, Igor Luiz Carvalho Máximo, Lucas Rannier Ribeiro Antonino Carvalho, Odrey Mesa Fleitas

**Affiliations:** ^1^Department of Animal Science, Universidade Federal Rural de Pernambuco, Recife, Brazil; ^2^Department of Animal Science, Universidade Federal da Paraíba, Areia, Brazil; ^3^Department of Physiology and Pharmacology, Stockholm Sweden Biomedicum, Karolinska Institutet, Stockholm, Sweden; ^4^Institute of Animal Science (ICA), Havana, Cuba

**Keywords:** alternative feed, carbohydrases, intestine, histology, non-starch polysaccharides

## Abstract

*Moringa oleifera* leaf meal (MOL) can be an alternative food in poultry diets. Still, their use is limited due to the presence of non-starch polysaccharides (NSP) and phytates. The supply of leaves associated with exogenous enzymes can influence the weight, size, and morphology of the intestine, accessory glands, and reproductive system of laying hens. The aim was, therefore, to study the influence of MOL in diets supplemented with and without the enzymes xylanase and phytase, whether associated or not, and their effects on the size and weight of the organs of the gastrointestinal tract, accessory glands, and reproductive system, as well as on the morphological characteristics of the small intestine of laying hens intended for egg production during the peak laying period. A total of 288 laying hens of the Dekalb White strain aged 32 weeks were used, distributed in a completely randomized design in a 2 × 4 factorial arrangement (presence and absence of MOL x 4 forms of enzyme supplementation - with or without) for a total of eight treatments with six replicates of six birds per experimental unit. The treatments consisted of a control diet based on corn and soybean meal and a diet with 5% MOL supplementation followed by three forms of enzyme supplementation (xylanase, phytase, and a mix of the two enzymes). Xylanase, alone or in combination with phytase, played an essential role in reducing the relative size of the small intestine and caeca, as well as improving intestinal morphology by increasing villus height, crypt depth, villus height/crypt depth ratio, mucosal length, and villus width, thus characterizing improvements in the processes of digestion and absorption of nutrients. Phytase has not influenced the variables studied in this study. Dietary inclusion of *Moringa oleifera* leaf (MOL) and exogenous enzymes, particularly xylanase, significantly influenced organ weights and intestinal morphology in laying hens. Xylanase, especially when combined with MOL, enhanced villus height, crypt depth, V:C ratio, and mucosal length across all intestinal segments, while phytase had more variable effects depending on diet context. It can be concluded that the NSP present in MOL have various effects on the intestine, such as increasing the size of the intestine and attached glands and influencing intestinal morphometric characteristics. With the degradation of these nutrients, the action of xylanase re-establishes the birds’ intestinal health, which could lead to better performance from the layers.

## 1 Introduction

Plants are the most widely used food sources in the formulation of poultry diets, with corn and soybean meal being these animals’ primary feedstuffs. Plant-based ingredients contain varying levels of non-starch polysaccharides (NSPs), which are structural components of the plant cell wall and primarily serve a protective function (1). These fiber compounds are composed of glycosidic bonds (*β*-1,4 and β-1,6) that cannot be broken down by the animal’s endogenous enzymes. However, they can be degraded by microbial enzymes produced by the gut microbiota (2, 3).

The effect of these compounds on the gastrointestinal tract (GIT) of poultry depends on the solubility of each molecule. Soluble NSP has a high-water retention capacity. They can increase the viscosity and volume of the digesta, decrease intestinal transit, as well as compromise the association of enzymes and substrates, impairing the digestion of proteins, fats, and carbohydrates (4–8). Insoluble NSP, on the other hand, speed up intestinal transit, reducing the time the digested remains in contact with endogenous digestive enzymes, as well as encapsulating nutrients within the plant cell, making them unavailable for use by poultry (2, 6, 9–11).

Herbaceous plants have received considerable attention for improving the performance and health status of commercial layers (12). In this context, *Moringa oleifera* leaves (MOL) show great potential for use in poultry nutrition due to their rich nutritional profile, including an average protein content of around 25%, as well as high levels of calcium, phosphorus, flavonoids, ascorbic acid, alpha-tocopherol, polyphenols, glycosides, and phenolic compounds. MOL, commonly referred to as the “miracle tree,” is recognized for its exceptional nutritional composition. In poultry nutrition, it has been explored as a natural feed additive due to its multiple beneficial effects. When included at optimal levels in broiler diets, MOL has been shown to improve body weight gain and feed conversion ratio. Additionally, its bioactive compounds help control pathogenic gut bacteria, contributing to improved intestinal health and a reduced need for antibiotic use. The inclusion of MOL also enhances nutrient digestibility. In laying hens, it has been associated with improvements in egg production and egg quality parameters, such as yolk pigmentation and shell thickness, likely due to its content of carotenoids and essential minerals (13–21).

However, thanks to the high NSP content present in this food, its use in poultry nutrition is still limited. Macambira et al. (22) found that most of the fibrous compounds in *Moringa oleifera* leaves belong to the soluble fraction. Formed by the hemicellulose fractions (xyloglucans, xylans, arabinoxylans, *β* glucans, among others), gums and pectins, these components have a high capacity for absorbing water, resulting in an increase in the viscosity and volume of the digestate, compromising the association of enzymes and substrates, reduced intestinal transit, changes in the secretion of pancreatic juice and other secretory mechanisms of the GIT, an increase in the size of the liver, pancreas, and intestines, thanks to the more significant presence of undigested substrate in the intestinal lumen (4, 6, 8, 23–25). In addition, they can compromise the intestinal morphological characteristics with reduced villus width and size, reduced crypt depth, and villus height/crypt depth ratio (23, 26–29). MOL also contains significant amounts of phytate, which complexes and make unavailable minerals, such as phosphorus, calcium, magnesium, iron, and zinc, and bind to proteins, fibers, and other nutrients.

Exogenous enzymes, mainly carbohydrates, and phytases, represent promising alternatives that allow greater use of fibrous foods in poultry diets (30–34). Although there are studies that have verified the influence of *Moringa oleifera* leaves on intestinal morphology in broiler chickens (18, 35, 36), no studies on this topic were found with commercial layers. On the other hand, we are unaware of any studies that have examined the effects of the association of carbohydrates in diets containing this vegetable on the morphological characteristics of the intestine in poultry. Therefore, the results of this study represent a new approach to using this promising plant species in poultry feeding for egg production.

The hypothesis was that dietary inclusion of *Moringa oleifera* leaves (MOL) at a 5% inclusion level, in combination with exogenous enzymes, would positively influence the morphological characteristics of the small intestine, the size of gastrointestinal organs and associated glands, as well as the reproductive system of laying hens during their peak laying period. The aim was, therefore, to study the influence of MOL in diets supplemented with and without the enzymes xylanase and phytase, whether or not associated, and their effects on the size and weight of the GIT organs, reproductive system, and attached glands, as well as on the morphological characteristics of the small intestine of laying hens destined for egg production during the peak laying period.

## 2 Materials and methods

The research was approved by the Ethics Committee on the Use of Animals (CEUA), of the Federal Rural University of Pernambuco, in accordance with license number 21/2018.

### 2.1 Production of *Moringa oleifera* leaf meal and bromatological analysis

*Moringa oleifera* leaves, and petioles collected 45 days apart were used to obtain the material which combine green matter production and the nutritional value of the leaves. The plants were cut at a height of approximately 60 cm from the ground. After harvesting, the plants were first dried in a shed until their weight stabilized and then chopped into fodder. The material was then ground in a vertical mill to obtain leaf meal.

Samples of MOL and experimental feed were collected and sent to the Animal Nutrition Laboratory (ANL) of the Animal Science Department of the Federal Rural University of Pernambuco (UFRPE) for determination of the dry matter (DM), crude protein (CP), ether extract (EE) and mineral matter (MM) contents, according to the methodologies proposed by Detmann et al. (37). Neutral detergent fiber (NDF) and acid detergent fiber (ADF) were determined using the method proposed by Van Soest (38). Gross energy (BE) was determined using a calorimetric pump (IKA, model C-200). The determined composition of the MOL is shown in Table 1.

**Table 1:** Chemical composition of *Moringa oleifera* leaf meal (in natural matter).

Nutrients	Total amino acids (%)^2^	
Dry matter, % 89.95	Methionine	0.324
Crude protein, % 20.23	Cystine	0.228
Neutral detergent fiber, % 39.76	Methionine + Cystine	0.554
Acid Detergent Fiber, % 19.11	Lysine	0.995
Mineral Matter, % 12.05	Threonine	0.822
Ether extract, % 8.43	Tryptophan	0.392
Gross energy (MJ/kg) 19.34	Arginine	1.058
Metabolizable energy (MJ/kg)^1^ 12.62	Isoleucine	0.822
	Leucine	1.595
	Valine	1.032
	Histidine	0.403
	Phenylalanine	0.999
	Glycine	0.956
	Serina	0.791
	Proline	0.917
	Alanine	1.159
	Aspartic Acid	1.634
	Glutamic acid	2.166
	Glycine + Serine	1.747
	Digestible amino acids (%)^3^	
	Methionine	0.251
	Lysine	0.617
	Methionine + Cystine	0.349
	Threonine	0.509
	Arginine	0.677

^1^Value estimated by Silva (41); ^2^Total AA values estimated by Macambira et al. (21); ^3^Values estimated considering digestibility of 62, 75, 63, 62 and 64%, respectively, for the amino acids Lysine, Methionine, Methionine + Cystine, Threonine and arginine in alfalfa (38).

### 2.2 Birds and facilities

The study used 288 laying hens of the Dekalb White strain aged 32 weeks with an average initial weight of 1,520 kg. They were housed in cages measuring 1.00 × 0.40 × 0.45 m, equipped with a trough for collecting eggs, a trough-type feeder, and an automatic drinker with an attached cup. The birds were weighed at the start of the experimental period to ensure uniformity between the experimental plots. The animals then had their egg production monitored, per experimental unit, for 14 days. Once the weight and egg production were uniform, the treatments were randomly distributed among the experimental units and reared for 18 weeks or 140 days.

**Figure 1:**
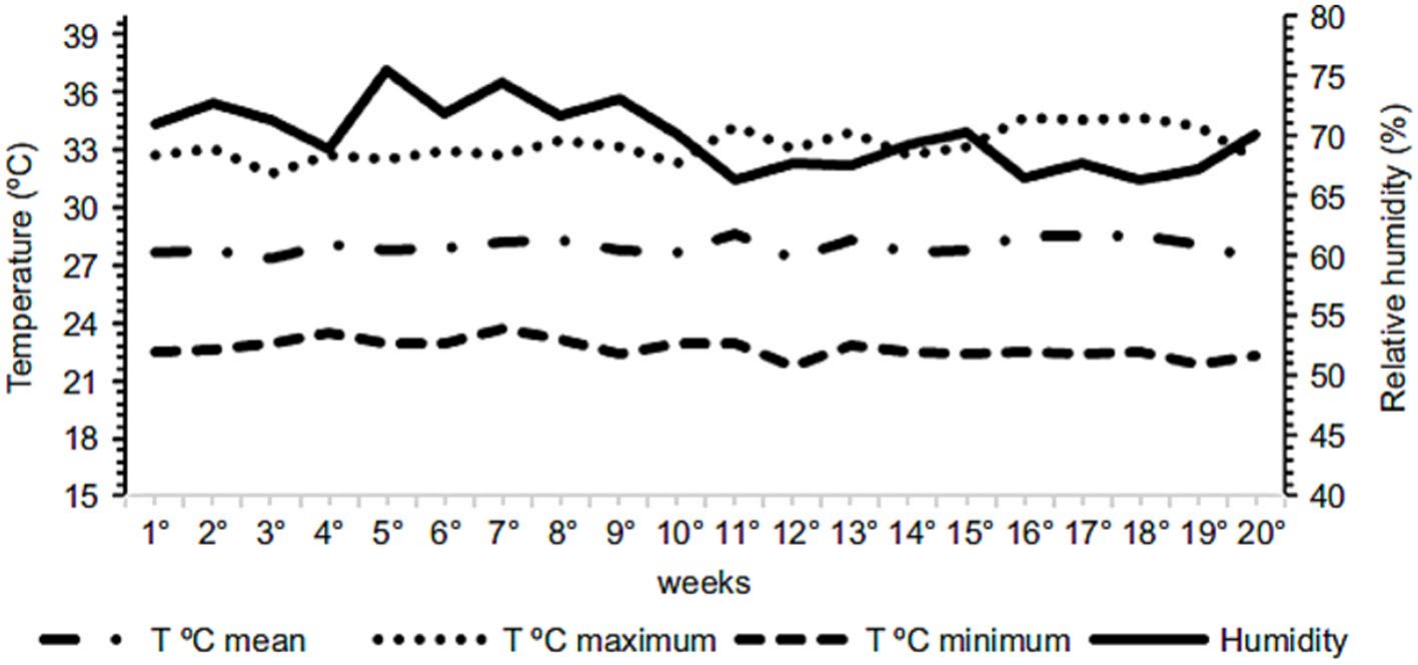
Variation in temperature and relative humidity during the experimental period.

The light program was adopted for 17 h, consisting of 12 h of natural light + 5 h of artificial light. The environmental parameters, temperature, and relative humidity were measured daily using a data logger (HOBO, model U12-001), as well as a thermohydrometer (Incoterm Digital, model 7666.02.0.00) installed in the middle of the house at the height of the birds’ backs, throughout the experiment. The average temperature and relative humidity during the experimental period were 25.79°C and 69.92%, respectively. The variations in temperature and relative humidity throughout the period are illustrated in Figure 1.

### 2.3 Design and experimental diets

The birds were distributed in a completely randomized design in a 2 × 4 factorial arrangement (supplementation or not of 5% *Moringa oleifera* leaf meal x four forms of enzyme supplementation – with or without) for eight treatments. The xylanase used was Econase XT 25P (AB Vista, Florida), a bacterial enzyme expressed in *Trichoderma* sp. with an activity of 160,000 BXU of endo 1,4-b-xylanase per gram. The supplemented phytase was Quantum-Blue 5 G (AB Vista, Florida), an enzyme isolated from *Escherichia coli* with an activity of 300FTU. A Beechwood Xylanase Unit (BXU) is the enzyme capable of releasing 1 nmol of birch xylan, measured in xylose equivalents, under assay conditions (AB Enzymes, Germany). The FTU, or active phytase unit, is the amount of enzyme required to release 1 μmol of inorganic phosphorus per minute from a substrate of 0.0051 mol/L of sodium phytate at pH 5.5 and 37°C (39).

The experimental treatments were as follows: a control diet without enzyme supplementation (C); a control diet supplemented with 75 g/tonne of xylanase (CX); a control diet supplemented with 60 g/tonne of phytase (CF); a control diet supplemented with both 75 g/tonne of xylanase and 60 g/tonne of phytase (CMIX); a diet containing 5% *Moringa* leaf meal without enzyme supplementation (M); a *Moringa*-based diet supplemented with 75 g/tonne of xylanase (MX); a Moringa-based diet supplemented with 60 g/tonne of phytase (MF); and a *Moringa*-based diet supplemented with both 75 g/tonne of xylanase and 60 g/tonne of phytase (MMIX). The lowest enzyme dosages recommended by the manufacturers were selected in order to evaluate the minimum effective inclusion level capable of promoting improvements in performance and nutrient utilization. This approach allows for a cost-effective assessment of enzyme efficacy, which is particularly relevant for practical applications in commercial poultry production. Additionally, using the lowest recommended dose helps minimize potential interactions or over-supplementation effects when combined with other dietary components such as *Moringa oleifera* leaf meal.

Table 2 shows the centesimal composition of the ingredients and the calculated and determined nutritional composition of the experimental rations. The rations were formulated according to the feed composition in the Brazilian Tables for Poultry and Pigs developed by Rostagno et al. (40), except for the MOL, which had its nutritional profile analyzed at the ANL. The apparent metabolizable energy content of 3,014 kcal/kg for MOL was determined by Silva (41) in a metabolism trial with layers. Reference AA data determined by Macambira et al. (22) and estimates of digestible AA considering a digestibility of 62, 75, 63, 62, and 64% for Lysine, Methionine, Methionine + Cystine, Threonine, and Arginine from alfalfa (42) were used to formulate the diets (Table 1). The manual for the breed used met all the birds’ nutritional requirements. The nutritional profile was evaluated for the diets containing the enzymes xylanase and phytase at 100 kcal and 0.15% of available phosphorus, respectively. To better observe their effect on the feed components, all the ingredients were kept stable, with only the amounts of soybean oil and calcium phosphate added varyingpna.

**Table 2:** Chemical composition and nutritional values of experimental diets.

Ingredients	Treatments							
	C	CX	CF	CXF	M	MX	MF	MXF
Corn	55.210	55.210	55.210 55,210	55.210	52.385	52.385	52.385	52.385
Soybean meal	28.390	28.390	28.390	28.390	26.235	26.235	26.235	26.235
Soybean oil	3.783	2.759	3.783	2.759	3.933	2.912	3.936	2.912
Bicalcium phosphate	2.280	2.349	1.732	1.732	2.275	2.343	1.727	1.727
Limestone	9.328	9.548	9.824	9.957	9.114	9.334	9.612	9.742
Common salt	0.261	0.311	0.311	0.311	0.267	0.317	0.317	0.317
DL-Methionine 99%	0.282	0.282	0.282	0.282	0.293	0.293	0.293	0.293
L-Lysine HCl 78.8%	0.043	0.043	0.043	0.043	0.070	0.070	0.070	0.070
Sodium bicarbonate	0.150	0.150	0.150	0.150	0.150	0.150	0.150	0.150
Vitamin Premix^1^	0.100	0.100	0.100	0.100	0.100	0.100	0.100	0.100
Mineral premix^2^	0.100	0.100	0.100	0.100	0.100	0.100	0.100	0.100
L-Threonine 98.5%	0.069	0.069	0.069	0.069	0.079	0.079	0.079	0.079
Xylanase	–	0.0075	–	0.0075	–	0.0075	–	0.0075
Phytase	–	–	0.0060 0,0060	0.0060	–	–	0.0060 0,0060	0.0060
*Moringa oleifera leaf meal* (MOL)	–	–	–	–	5.000	5.000	5.000	5.000
Inert	–	0.681	–	0.883	–	0.684	–	0.886
Calculated nutritional composition								
Metabolizable energy (kcal/kg)	2,850	2,850	2,850	2,850	2,850	2,850	2,850	2,850
Crude protein (%)	17.500	17.500	17.500	17.500	17.500	17.500	17.500	17.500
Fiber (%)	2.758	2.757	2.757	2.757	3.361	3.360	3.360	3.360
Calcium (%)	4.300	4.300	4.249	4.300	4.300	4.300	4.300	4.300
Sodium (%)	0.180	0.200	0.200	0.200	0.180	0.200	0.200	0.200
Available phosphorus (%)	0.420	0.430	0.430	0.430	0.420	0.430	0.430	0.430
Digestible lysine (%)	0.860	0.860	0.860	0.860	0.860	0.860	0.860	0.860
Digestible tryptophan (%)	0.201	0.200	0.200	0.201	0.186	0.186	0.186	0.186
Digestible threonine (%)	0.660	0.660	0.660	0.660	0.660	0.660	0.660	0.660
Digestible methionine + cystine (%)	0.757	0.757	0.757	0.757	0.757	0.757	0.757	0.757
Digestible leucine (%)	1.391	1.391	1.391	1.391	1.297	1.300	1.300	1.300
Digestible Valine (%)	0.711	0.711	0.711	0.711	0.661	0.661	0.661	0.661
Nutritional composition analyzed								
Dry matter (DM) (%)	90.347	90.125	90.544	90.337	91.670	91.021	91.665	91.001
Crude protein (CP) (%)	17.432	17.578	17.521	17.415	17.512	17.585	17.599	17.545
Neutral detergent fiber (NDF) (%)	23.555	23.461	23.530	23.345	25.407	25.411	25.400	25.378
Acid detergent fiber (ADF) (%)	6.210	6.256	6.195	6.188	7.399	7.369	7.421	7.306
Ash (%)	16.000	16.007	15.832	15.760	16.242	16.198	15.926	15.934

^1^Vitamin premix guarantee levels: vitamin A (min): 9,000,000 IU/kg, vitamin D3 (min): 1,600,000 IU/kg, vitamin E (min): 14,000 IU/kg, vitamin K3 (min) 1,500 mg/kg, vitamin B1 (min): 1,000 mg/kg, vitamin B (min): 4,000 mg/kg, vitamin B6 (min): 1,800 mg/kg, vitamin B 12 (min): 12.000mcg/kg, folic acid (min): 300 mg/kg, pantothenic acid (min): 8,280 mg/kg, biotin (min): 50 mg/kg, niacin (min): 30 g/kg, selenium (min): 250 mg/kg. ^2^Mineral premix guarantee levels: iron (min): 60 g/kg, copper (min): 18 g/kg, manganese (min): 120 g/kg, and zinc (min):120 g/kg.

### 2.4 Organs of the gastrointestinal tract and productive system

At the end of the experimental period, twelve birds per treatment (two per experimental unit, with an average weight within the same unit) were euthanized by cervical dislocation and sent to the Meat Laboratory of the Animal Science Department at UFRPE to measure the weight of the organs of the gastrointestinal tract (liver, gizzard, small intestine, large intestine, cecum, and pancreas), reproductive system (ovary and oviduct) and spleen, as well as the length of the intestines (small, large and caeca). A 0.01 g precision scale was used to obtain the weight data, while a tape measure was used for the length measurements. Weight results were expressed in absolute weight (g) and length measurements (cm).

### 2.5 Intestinal histology

At the end of the experimental period, two birds per experimental unit (twelve per treatment), different from those used to assess weight and organ length, were euthanized by cervical detachment. Samples of 4 cm from sections of the small intestine (duodenum, jejunum, and ileum), liver, pancreas, and spleen were collected, washed with saline solution, and immersed in 10% buffered formalin. After these, the tissues were dehydrated in a series of alcohols with increasing concentrations (10, 80, 90, and 100%), immersed in xylene, and embedded in paraffin.

The height of the villi was measured from their apex to their base, while the depth of the crypt was measured from the crypt’s base to the villus’s base. The villus-crypt ratio was calculated as the ratio between villi and crypts. For each segment and variable analyzed, 20 measurements were taken, totaling 40 per experimental unit.

### 2.6 Statistical analyses

Each cage, housing six birds, was considered an experimental unit. For each treatment, six cages were used, resulting in a total of 48 experimental units across all treatments. The data was analyzed for homoscedasticity and homogeneity of variances. One-way ANOVA was conducted using the GLM procedure in SAS 9.4 (SAS Institute Inc., 2012) (43). The influence of the individual factors and interactions on the variables was analyzed. In the presence of significant differences, the means were compared using the Tukey test at 5% probability. The equation below shows the statistical model.


Yijk=μ+αi+βj+ðαβÞij+εijk

in which:

Yijk is the observed value, μ is the population average, αi is the effect of the MOL (1–2), βj is the enzymatic supplementation effect (1–4), (αβ)ij is the interaction effect between MOL and enzymatic supplementation, and εijk is the residual error.

## 3 Results

### 3.1 Organs of the gastrointestinal tract, accessory glands and reproductive system

According to Table 3, a significant interaction was observed between the experimental factors for liver and pancreas weights, as well as for the lengths of the small intestine and caeca. For gizzard weight, only a main effect of MOL inclusion was detected, with birds fed diets containing 5% MOL exhibiting significantly heavier gizzards. No significant effects (*p* > 0.05), either individual or interactive, were found for the weights of the small intestine, large intestine, caeca, and spleen, nor for the length of the large intestine.

**Table 3:** Effects of individual factors and unfolding for the variables weight and length of the organs of the digestive and reproductive systems of laying hens fed diets containing *Moringa* and supplemented with enzymes.

*Moringa*	Enzyme	Liver (g)	ID (g)	GI (g)	Cecos (g)	ID (cm)	IG (cm)	Cecos (cm)	Pancreas (g)	Gizzard (g)	Ovary (g)	Oviduct (g)	Spleen (g)
5% MOL		14.591	21.130	6.906	4.576	121.250	25.517	16.000	1.414	11.422^a^	18.522	26.652	1.314
0% MOL		14.737	19.374	6.920	4.550	130.175	24.308	17.100	1.207	9.508^b^	19.763	27.876	1.298
	Without	15.063	20,441	6.280	4.528	134.083	25.167	17.167^A^	1.147^B^	9.828	19.902	27.135	1.538
	Xylanase	14.460	19,889	5.912	3.552	121.633	25.117	14.801^B^	2.747^A^	10.097	19.534	25.615	1.594
	Phytase	13.802	21,850	6.496	4.872	124.400	25.500	18.300^A^	1.146^B^	10.713	19.575	25.619	1.439
	Mix	12.332	19,329	5.759	3.895	122.733	23.566	14.783^B^	2.704^A^	10.181	19.564	26.156	1.515
Developments													
5% MOL	Without enzyme	15.292^Aa^	19.010	6.898	4.838	133.000^Aa^	25.667	18.500^Aa^	3.012^Aa^	10.802	20.680	25.563	1.557
	Xylanase	12.868^Ab^	18.777	6.045	3.995	108.600^Bc^	24.400	14.102^Bb^	1.830^Ab^	12.036	20.968	25.680	1.613
	Phytase	14.443^Aa^	21.242	7.566	4.851	123.600^Ab^	26.600	18.200^Aa^	3.182^Aa^	11.058	19.880	25.266	1.522
	Mix	11.368^Bb^	18.468	5.615	3.912	109.800^Bc^	25.400	14.042^Bb^	1.830^Ab^	11.062	20.572	25.200	1.504
0% MOL	Without enzyme	15.833^Aa^	21.873	6.661	4.218	135.166^Aa^	24.667	17.722^Aa^	1.193^Ba^	8.936	20.124	25.708	1.520
	Xylanase	13.053^Aa^	21.000	6.290	3.983	124.667^Ab^	25.883	16.123^Ab^	1,101^Ba^	9.427	19.830	26.616	1.613
	Phytase	15.172^Aa^	21.458	6.425	4.888	135.000^Aa^	24.400	18.301^Aa^	1.110^Ba^	10.368	20.270	25.972	1.522
	Mix	13.305^Ab^	20.190	5.904	4.141	125.667^Ab^	22.333	16.012^Aa^	1.226^Ba^	9.200	20.555	25.616	1.613
Sources of variation	*p*-value												
*Moringa*		NS	NS	NS	NS	NS	NS	NS	NS	**0.019**	NS	NS	NS
Enzyme		NS	NS	NS	NS	**0.043**	NS	**0.003**	**0.032**	NS	NS	NS	NS
*Moringa* x Enzyme		**0.043**	NS	NS	NS	**0.015**	NS	**0.023**	**0.049**	NS	NS	NS	NS

*In the individual factors, lowercase and uppercase letters compare the means within the enzyme or *Moringa* factor, respectively. Means followed by the same letter in the column differ statistically by the Tukey test at 5% probability. In the breakdowns, capital letters compare means between the “No *Moringa*” and “*Moringa*” groups; lower case letters compare means within the group itself. Values followed by different upper and lower case letters in the column do not differ statistically within and between the groups, respectively, according to the Tukey test at 5% probability. The bold values indicate significant values.

**Figure 2:**
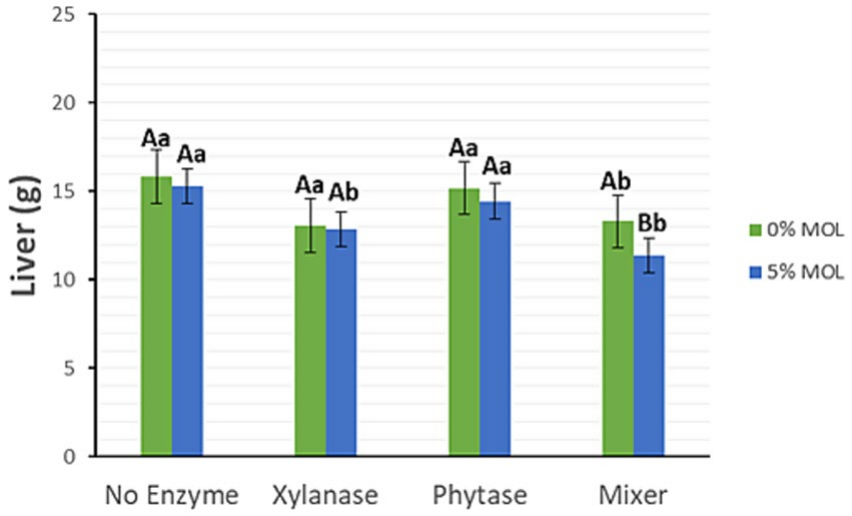
MOL x enzyme interaction for liver weight. Upper-case letters compare means between the “0% MOL” and “5% MOL” groups, lower case letters compare means within itself.

As shown in Figure 2, dietary supplementation with xylanase, either alone or in combination with phytase, significantly reduced liver weight (*p* < 0.05) in the presence of MOL. In the absence of MOL, individual enzyme supplementation had no significant effect on liver weight; however, the combined use of xylanase and phytase tended to reduce liver weight (*p* < 0.05).

**Figure 3:**
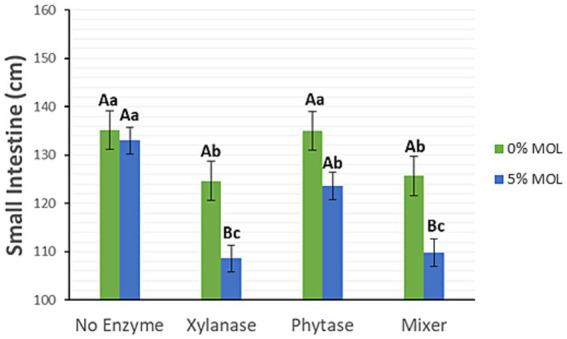
MOL x enzyme interaction for small intestine length. Upper-case letters compare means between the “0% MOL” and “5% MOL” groups, and lowercase letters compare means within the group itself.

**Figure 4:**
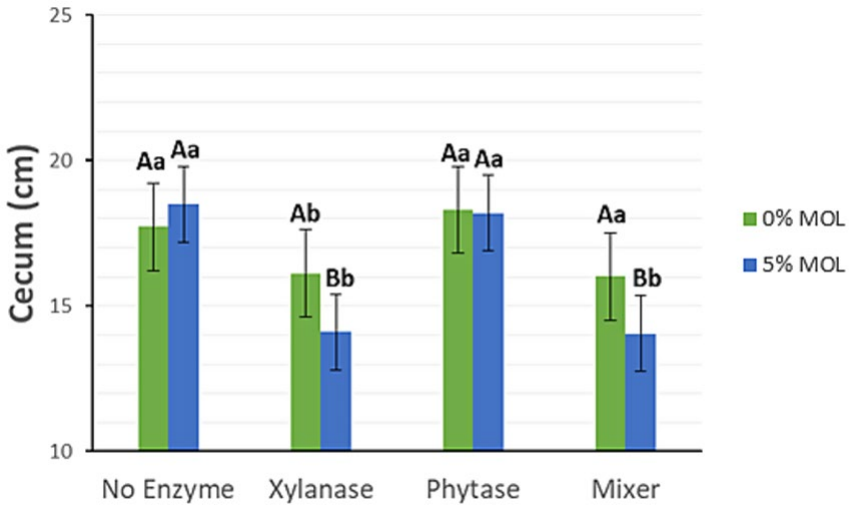
MOL x enzyme interaction for cecum length. Upper-case letters compare means between the “0% MOL” and “5% MOL” groups; lower case letters compare means within the group.

Xylanase supplementation in the presence of MOL, whether administered individually or in combination with phytase, significantly reduced the length of the small intestine (*p* < 0.05), as illustrated in Figure 3. Birds fed diets without enzyme supplementation, regardless of MOL inclusion, exhibited longer intestines. Diets containing phytase alone also resulted in longer intestines. A similar pattern was observed for caecal length (*p* < 0.05) (Figure 4).

**Figure 5:**
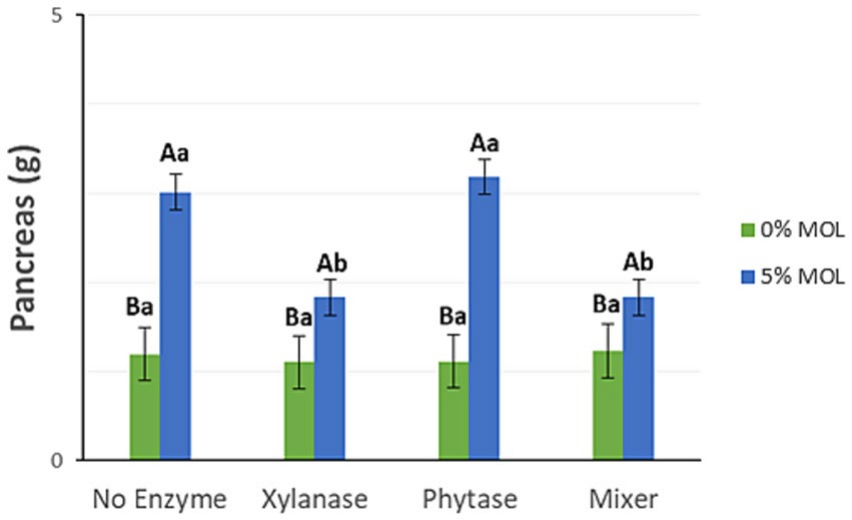
MOL x enzyme interaction for pancreas weight. Upper-case letters compare means between the “0% MOL” and “5% MOL” groups, lower case letters compare means within the group itself.

Figure 5 shows that the inclusion of MOL significantly increased pancreas weight. However, laying hens receiving MOL-supplemented diets along with xylanase—either alone or in combination with phytase—had significantly lower pancreas weights compared to those fed diets without enzymes or with phytase alone (*p* < 0.05).

### 3.2 Intestinal morphology

Table 4 shows the intestinal morphology results of laying hens fed diets containing MOL supplemented with exogenous enzymes. The three intestinal segments analyzed had an interaction effect for all the variables studied.

**Table 4:** Effects of individual factors and unfolding for the variables weight and length of the organs of the digestive and reproductive systems of laying hens fed diets containing *Moringa* and supplemented with enzymes.

*Moringa*	Enzyme	Duodenum					Jejunum					ileum				
		VH (μm)	C (μm)	V:C (μm)	ML (μm)	VW (μm)	VH (μm)	C (μm)	V:C (μm)	ML (μm)	VW (μm)	VH (μm)	C (μm)	V:C (μm)	ML (μm)	VW (μm)
5% MOL		2187a	317.4a	7.2	2.505	316.6	3055a	424.0a	7.5b	3479a	373.8a	3810a	468.9	8.5a	4281a	415.0a
0% MOL		2112b	292.9b	7.5	2.426	293.3	2716b	361.1b	8.0a	3077b	334.8b	3460b	467.6	7.5b	3935a	373.2b
	No enzyme	1660B	265.0B	6.6B	1923B	279.8C	2629B	350.6B	7.9A	2980B	325.2B	3155C	412.9C	8.0B	3571C	361.8B
	Xylanase	2631A	375.9A	8.7A	2946A	310.0B	3116A	416.7A	7.7B	3533A	310.3B	4053A	473.5B	8.7A	4532A	418.8A
	Phytase	1655B	259.8B	6.6B	1916B	266.4C	2625B	363.1B	7.9A	2988B	335.1B	3393B	455.2B	7.5C	3865B	381.7B
	Mix	2653A	379.8A	8.5A	3034A	363.7A	3171A	439.8A	7.5C	3611A	416.6A	3939A	525.1A	7.7C	4464A	411.5A
Developments																
5% MOL	No enzyme	1585Bc	248.8Bc	6.6Ab	1834Bb	267.5Bc	2745Ab	402.6Ac	7.0Bb	3147Ab	330.2Ac	3.371.0Ab	382.4Bc	7.3Ac	3764Ab	359.7Ab
	Xylanase	2754Aa	431.1Aa	6.2Ab	3065Aa	417.5Aa	3311Aa	414.2Ab	8.1Aa	3725Aa	459.8Aa	4.299.0Aa	474.4Aab	9.3Aa	4784Aa	461.5Ab
	Phytase	1660Ab	261.9Ab	6.5Bb	1922Ab	263.1Ac	2729Ab	425.8Aab	6.8Bb	3155Ab	395.0Ab	3294Bb	436.2Bb	7.8Ab	3731Bb	376.2Ab
	Mix	2751Aa	447.3Aa	8.6Ba	3198Aa	418.5Aa	3435Aa	453.4Aa	7.8Aa	3888Aa	450.3Aa	4277Aa	569.0Aa	9.7Aa	4846Aa	466.4Aa
0% mol	No enzyme	1734Ab	281.2Ab	6.6Ab	2012Ab	292.1Aa	2514Bb	298.6Bb	8.8Aa	2813Bb	320.3Ab	2939Bc	438.4Ab	6.9Bc	3378Bc	364.3Ab
	Xylanase	2507Ba	320.4Ba	8.2Ba	2828Ba	302.5Ba	2921Ba	419.2Aa	7.4Bb	3340Ba	380.7Ba	3607Ba	472.8Aa	8.2Ba	4279Ba	383.3Ba
	Phytase	1650Ab	257.7Ac	6.7Ab	1910Ab	269.6Ab	2521Bb	300.4Bb	8.8Aa	2821Bb	335.3Bb	3492Ab	483.6Aa	7.2Bc	4000Ab	387.3Aa
	Mix	2557Ba	318.4Ba	8.50Aa	2869Ba	309.0Ba	2908Ba	426.3Ba	7.1Bb	3334Ba	382.9Ba	3601Ba	481.1Ba	7.8Bb	4082Ba	356.6Bb
Sources of variation	*p*-value															
*Moringa*		<0.001	NS	NS	NS	NS	<0.001	<0.001	<0.01	<0.001	<0.001	<0.001	<0.001	<0.01	<0.001	<0.001
Enzyme		<0.001	<0.001	<0.001	<0.001	<0.001	<0.001	<0.001	<0.01	<0.001	<0.001	<0.001	<0.001	<0.01	<0.001	<0.001
*Moringa* x Enzyme		<0.001	<0.001	<0.001	<0.001	<0.001	<0.001	<0.001	<0.01	<0.001	<0.001	<0.001	<0.001	<0.01	<0.001	<0.001

*In the individual factors, lowercase and uppercase letters compare the means within the enzyme or *Moringa* factor, respectively. Means followed by the same letter in the column differ statistically by the Tukey test at 5% probability. In the breakdowns, capital letters compare means between the “No *Moringa*” and “*Moringa*” groups, lower case letters compare means within the group itself. Values followed by different upper and lower case letters in the column do not differ statistically within and between the groups, respectively, according to the Tukey test at 5% probability.

### 3.3 Duodenum

**Figure 6:**
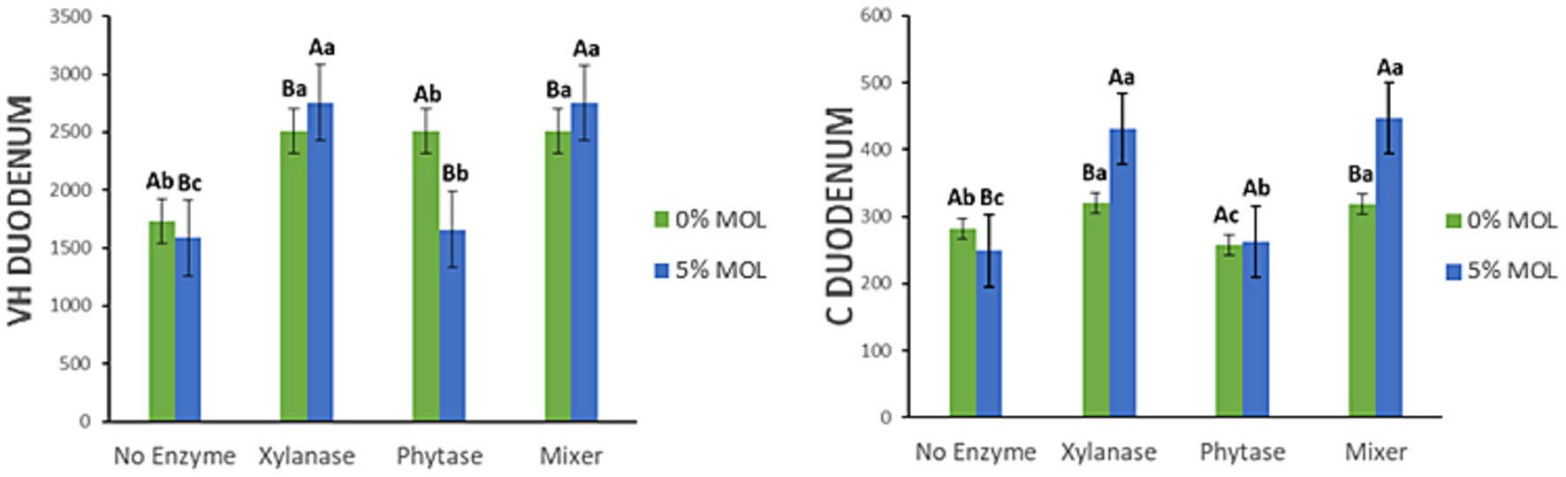
MOL x enzyme interaction for VH and C of the duodenum. Upper-case letters compare means between the “0% MOL” and “5% MOL” groups; lower case letters compare means within the group.

Figure 6 presents the interaction effects between *Moringa oleifera* leaf meal (MOL) inclusion and exogenous enzyme supplementation on villus height (VH) and crypt depth (C) in the duodenum of laying hens. The addition of xylanase to MOL-containing diets significantly increased duodenal villus height, both when xylanase was used alone and when combined with phytase (*p* < 0.05). In contrast, phytase alone reduced VH in MOL-based diets (*p* < 0.05). Interestingly, the opposite was observed in the control (non-MOL) diets: phytase supplementation in this case increased VH (*p* < 0.05). A similar interaction pattern was found for crypt depth. Xylanase, whether used alone or in combination with phytase, significantly increased crypt depth (*p* < 0.05). In diets without MOL, xylanase also increased VH and C, though the effect was less pronounced than in the MOL-based diets (*p* < 0.05).

**Figure 7:**
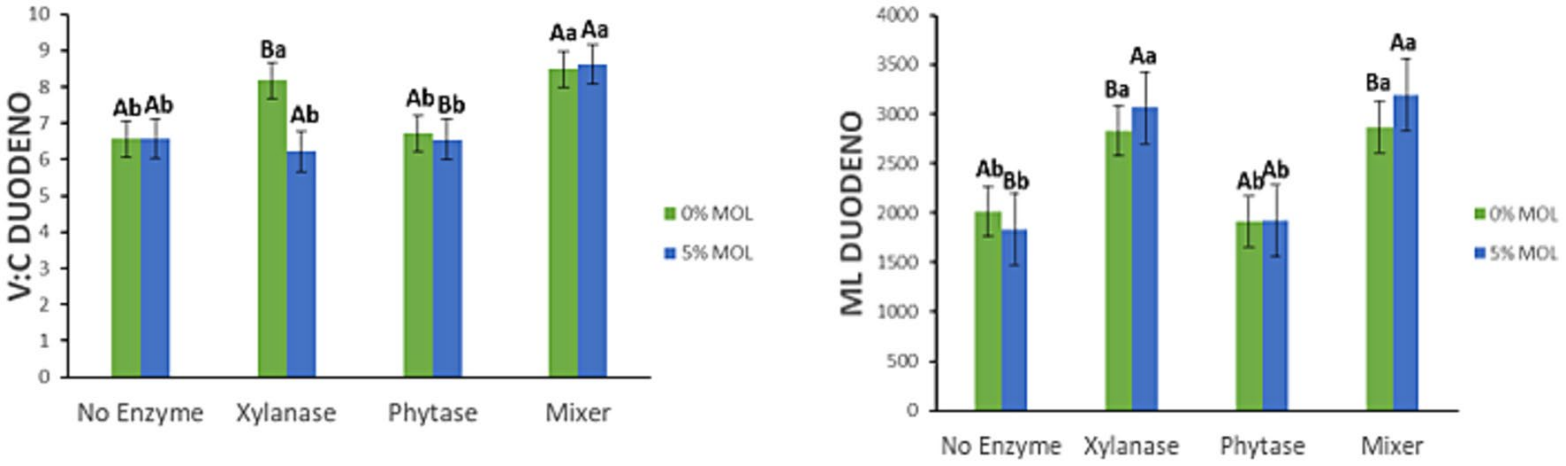
MOL x enzyme interaction for V:C and ML of the duodenum. Upper-case letters compare means between the “0% MOL” and “5% MOL” groups; lower case letters compare means within the group.

Figure 7 displays the interaction effects on the villus height-to-crypt depth ratio (V:C ratio) and mucosal length (ML) in the duodenum. The V:C ratio was significantly higher (*p* < 0.05) in hens fed MOL diets supplemented with the enzyme combination (xylanase + phytase), compared to those receiving only one of the enzymes. However, this increase was not significantly different from that observed in the control diet also supplemented with the enzyme mix (*p* > 0.05). Control diets supplemented with either xylanase or phytase individually also showed a significant increase in the V:C ratio (*p* < 0.05). Additionally, mucosal length was significantly greater in hens receiving MOL-based diets supplemented with xylanase, either alone or with phytase (*p* < 0.05). A similar but less marked increase in ML was observed in the control diets with xylanase (*p* < 0.05), while phytase alone had no significant effect on ML in either dietary context (*p* > 0.05).

**Figure 8:**
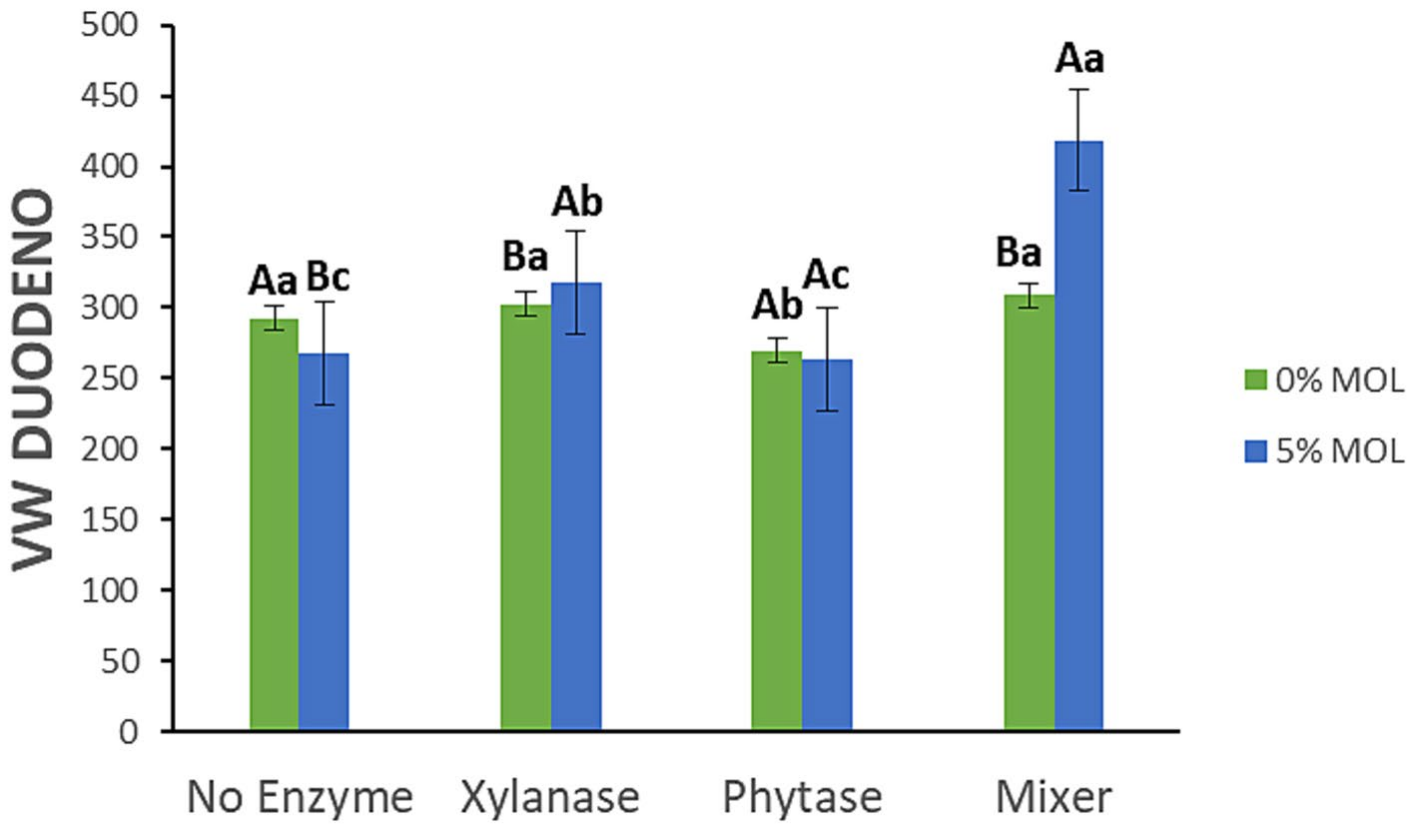
MOL x enzyme interaction for the VW of the duodenum. Upper-case letters compare means between the “0% MOL” and “5% MOL” groups; lower case letters compare means within the group.

Figure 8 highlights the interaction effects on villus width (VW) in the duodenum. Birds fed MOL diets supplemented with xylanase—alone or in combination with phytase—had significantly wider villi (*p* < 0.05). Phytase on its own had no significant effect on VW, and birds fed MOL without any enzyme supplementation exhibited narrower villi.

### 3.4 Jejunum

**Figure 9:**
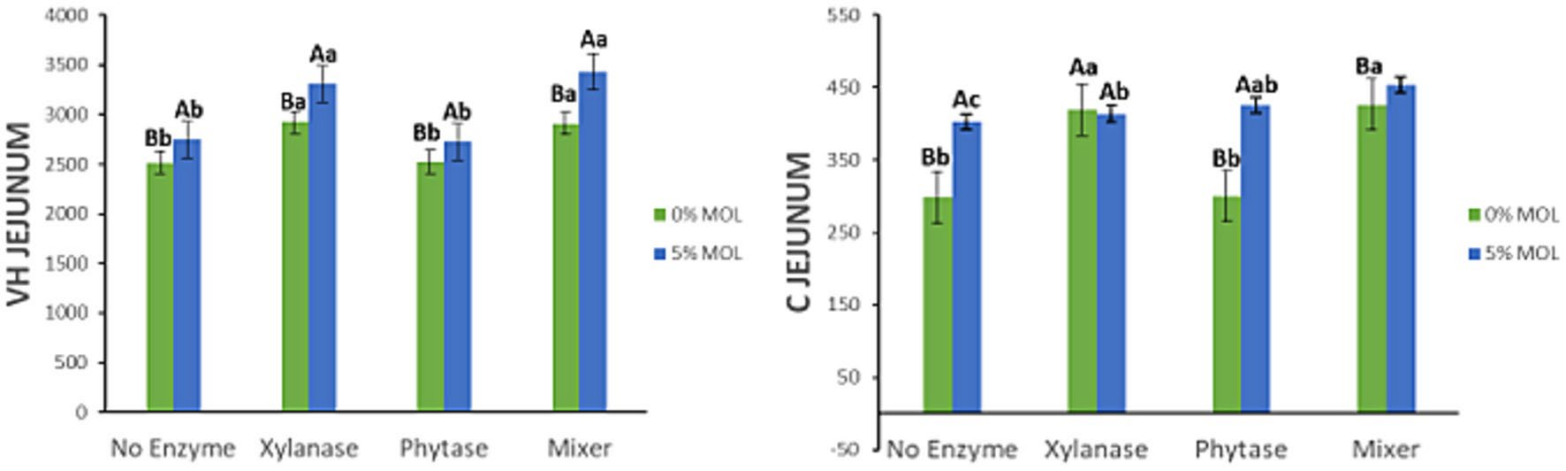
MOL x enzyme interaction for jejunal VH and C. Upper-case compare means between the “0% MOL” and “5% MOL” groups; lower case letters compare means within the group.

Figure 9 illustrates the interaction effects between *Moringa oleifera* leaf meal (MOL) inclusion and exogenous enzyme supplementation on villus height (VH) and crypt depth (C) in the jejunum of laying hens. In MOL-based diets, xylanase supplementation significantly increased VH (*p* < 0.05), with no notable difference between xylanase alone and its combination with phytase. Phytase alone also improved VH (*p* < 0.05), although the effect was less pronounced compared to xylanase. Interestingly, MOL inclusion without any enzyme supplementation significantly increased both VH and crypt depth (*p* < 0.05), indicating its inherent positive effect on intestinal morphology. The greatest crypt depth was observed in birds fed MOL in combination with the enzyme mix (*p* < 0.05), followed by those receiving phytase alone. However, crypt depth values in the xylanase-supplemented groups (alone or in combination) were not statistically different from the phytase group.

**Figure 10:**
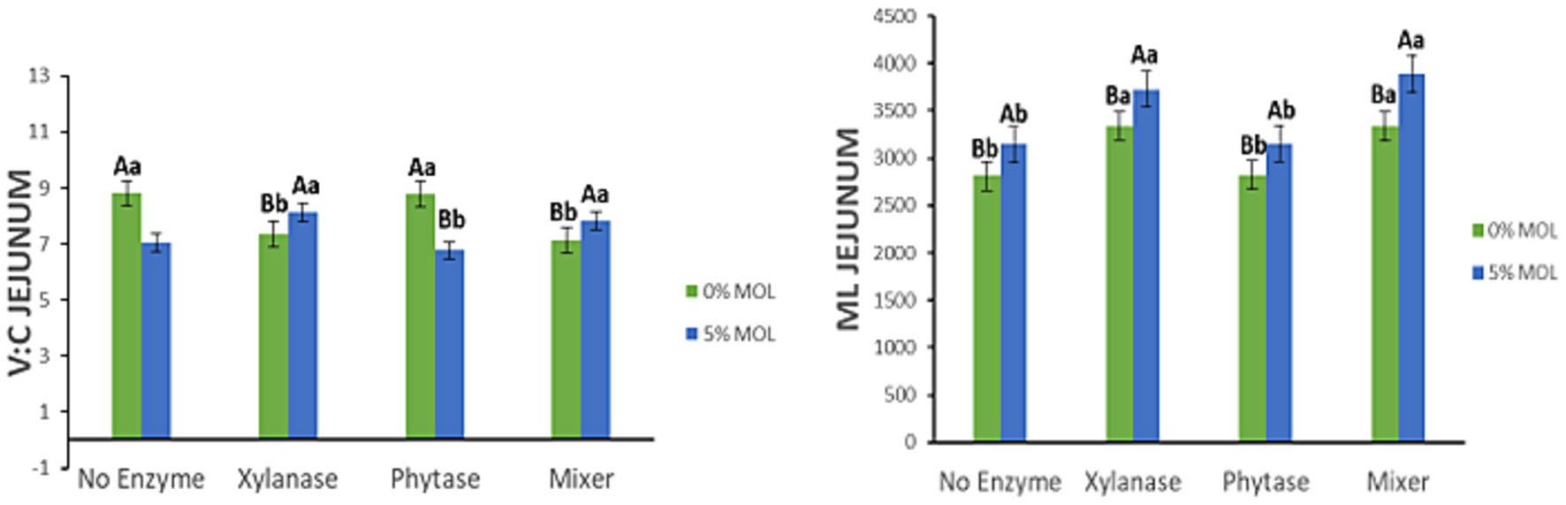
MOL x enzyme interaction for V:C and ML of the jejunum. Upper-case letters compare means between the “0% MOL” and “5% MOL” groups; lower-case letters compare means within the group.

Figure 10 presents the interaction effects on the villus height-to-crypt depth ratio (V:C) and mucosal length (ML) in the jejunum. Diets containing MOL supplemented with xylanase, whether alone or in combination with phytase, significantly increased the V:C ratio (*p* < 0.05). In contrast, lower V:C ratios were observed in birds fed phytase alone or MOL alone (*p* < 0.05), suggesting that xylanase plays a more prominent role in enhancing intestinal morphology. Similarly, mucosal length was significantly greater in hens fed MOL diets supplemented with xylanase (*p* < 0.05). Although phytase and MOL alone also contributed to increased ML, the effect was comparatively modest.

**Figure 11:**
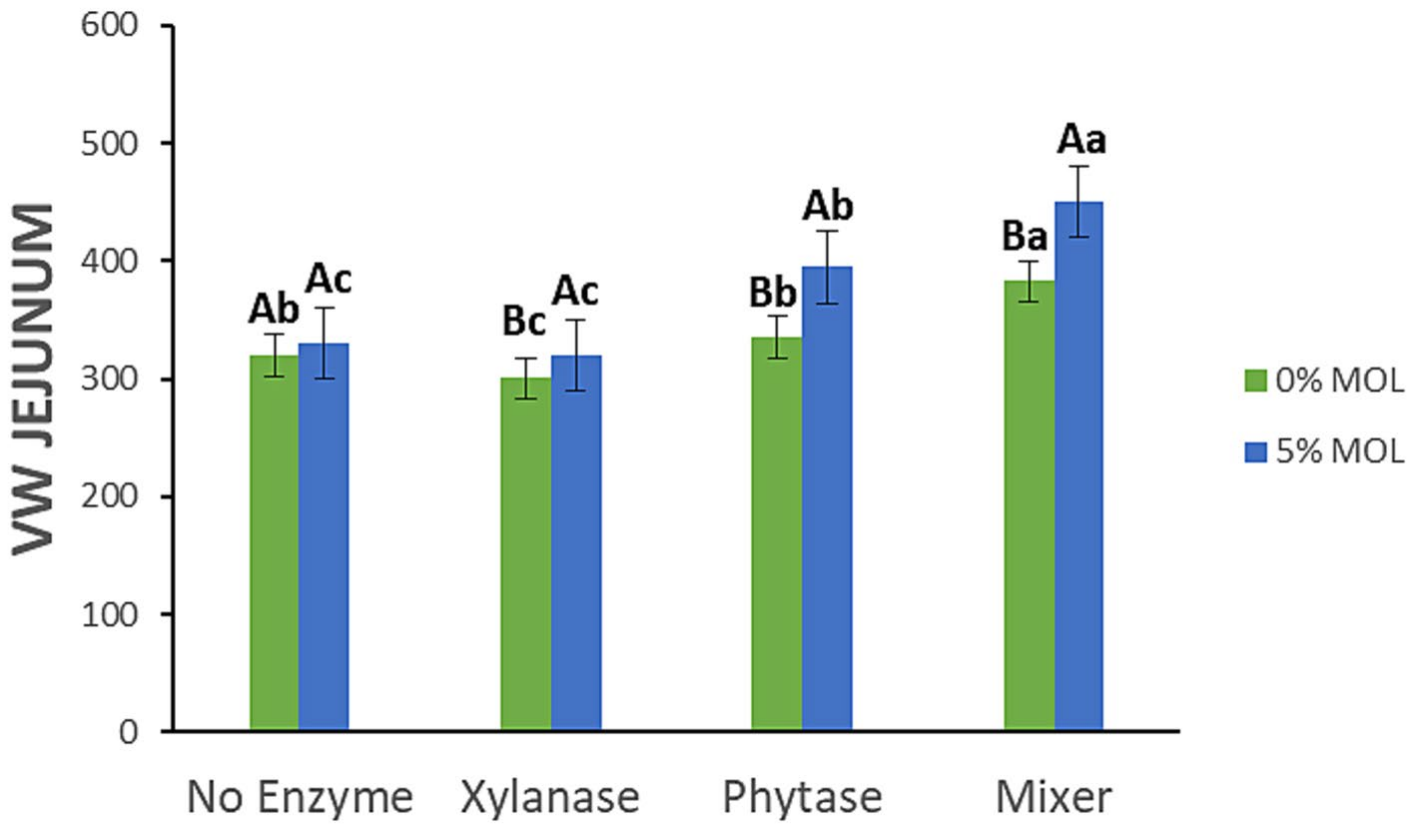
MOL x enzyme interaction for jejunal VW. Upper-case letters compare means between the “0% MOL” and “5% MOL” groups; lower case letters compare means within the group.

Figure 11 shows the interaction effects on villus width (VW) in the jejunum. Birds fed MOL diets supplemented with xylanase, either alone or in combination with phytase, exhibited significantly wider villi (*p* < 0.05). No significant differences in VW were observed among birds fed diets without enzyme supplementation, indicating that xylanase was the primary contributor to increased villus width in these treatments.

### 3.5 Ileum

**Figure 12:**
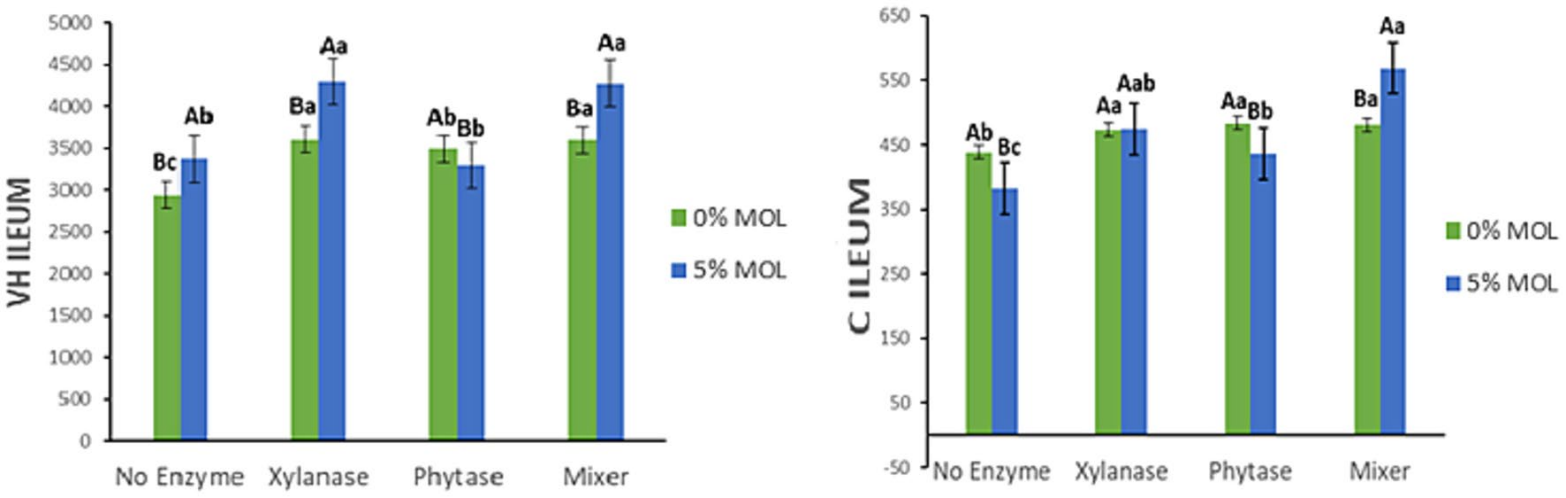
MOL x enzyme interaction for VH and C of the ileum. Upper-case letters compare means between the “0% MOL” and “5% MOL” groups; lower-case letters compare means within the group.

Figure 12 shows the interaction effects of *Moringa oleifera* leaf meal (MOL) and exogenous enzymes on villus height (VH) and crypt depth (C) in the ileum of laying hens. Birds fed diets containing MOL with xylanase—either alone or combined with phytase—exhibited significantly taller ileal villi (*p* < 0.05). In contrast, phytase supplementation in MOL diets was associated with a significant reduction in villus height (*p* < 0.05). Notably, MOL inclusion without enzyme supplementation also significantly increased villus height (*p* < 0.05). Regarding crypt depth, the enzyme combination (MIX) produced the deepest ileal crypts (*p* < 0.05), while phytase alone and MOL alone were linked to a decrease in crypt depth. Xylanase alone did not significantly affect crypt depth in the ileum (*p* > 0.05).

**Figure 13:**
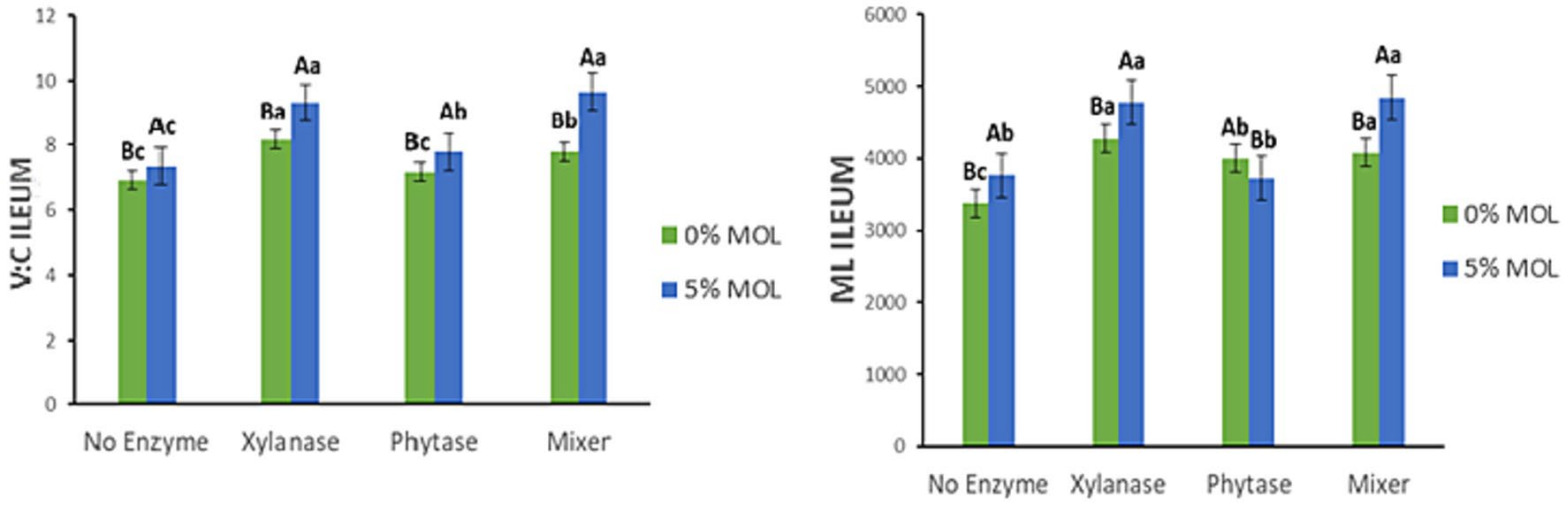
MOL x enzyme interaction for V:C and ML of the ileum. Upper-case letters compare means between the “0% MOL” and “5% MOL” groups; lower-case letters compare means within the group.

Figure 13 presents the interaction effects on the villus height-to-crypt depth ratio (V:C) and mucosal length (ML) in the ileum. Birds receiving MOL diets supplemented with xylanase, either individually or combined with phytase, had significantly higher V:C ratios (*p* < 0.05). Phytase and MOL alone also increased the V:C ratio but to a lesser extent (*p* < 0.05). A similar pattern was observed for mucosal length: xylanase supplementation in MOL diets significantly increased ileal mucosal length (*p* < 0.05). Conversely, phytase alone reduced mucosal length, while MOL alone increased it when no enzymes were included (*p* < 0.05).

**Figure 14:**
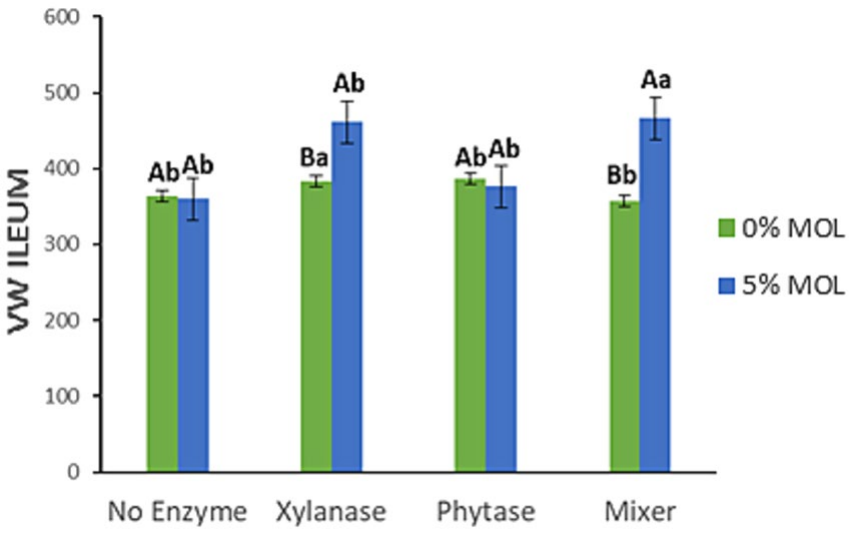
MOL x enzyme interaction for the VW of the ileum. Upper-case letters compare means between the “0% MOL” and “5% MOL” groups, and lower-case letters compare means within the group.

Figure 14 illustrates the interaction effects on villus width (VW) in the ileum. Significant increases in villus width were observed in birds fed MOL combined with xylanase, whether supplemented individually or in combination with phytase (*p* < 0.05). Neither phytase nor MOL alone had a significant effect on villus width in this intestinal segment (*p* > 0.05).

## 4 Discussion

The liver is the largest internal organ in the body, accounting for around 3% of a chicken’s body weight, and its size is associated with the age and body condition of the animal. In this study, the average body weight of the chickens was 1.725 kg (20). Research has shown that *Moringa oleifera* leaves possess antioxidant activity due to the presence of compounds such as flavonoids, ascorbic acid, alpha-tocopherol, beta-carotene, polyphenols, thiocarbons, glycosides, and phenolic compounds, which can serve as preventive agents against liver damage (13–15, 19, 20). Although some studies have reported an increase in liver weight in poultry fed *Moringa oleifera* leaf meal (MOL) (44), most research has found no significant effect of MOL on the relative liver size or function in broilers and laying hens (20, 45–47). These previous findings contrast with the results of the present study, which observed a significant influence of MOL on relative liver weight. The observed reduction in liver size with the combination of xylanase and *Moringa oleifera* may be explained by improved nutrient digestibility and absorption, leading to decreased metabolic stress on the liver. Furthermore, the antioxidant and anti-inflammatory compounds in MOL could protect liver tissue by preventing inflammation and fat accumulation, which are factors that contribute to liver enlargement. Regarding the pancreas, previous research has shown that *Moringa oleifera* leaves can increase its size, mainly due to the presence of bioactive compounds (as previously mentioned), which enhance pancreatic activity and inhibit the growth of pathogenic microbes in the intestines of birds, thereby positively affecting chicken metabolism and nutrition (48, 49).

On the other hand, the NSP in corn, soybean meal, and MOL may explain the decreases observed in the size of the liver and pancreas and the length of the small intestine and caeca of the birds when xylanase was supplemented. The basic composition of NSP includes cellulose, hemicellulose, xylans, and arabinoxylans, among others (2, 3). According to Bach Knudsen (50), total NSP in corn and soybean meal are approximately 9.70 and 21.70%, respectively. In contrast, the values reported for MOL by Macambira et al. (22) show that most of the NSP fraction in the leaves belongs to the soluble fiber fraction, as observed in this study, and that a considerable amount of the fiber in MOL is hemicellulose and its constituents. NSP are recognized for their ability to increase the weight and length of the intestine in birds (51, 52). Generally, these animals respond quickly to changes in the fiber content of the feed, showing modifications in intestinal size and passage rate. Amerah et al. (53) observed an increase in the length of the small intestine of broiler chickens fed whole wheat compared to those fed insoluble NSP sources. According to Khan et al. (35), the increase observed in the length of the intestine of broiler chickens fed MOL is due to increased digesta residence time in the gastrointestinal tract induced by the high fiber content of the feed. Although no significant differences were found in the weight of the small and large intestines in this study, the increases in the size of the small intestine and caeca in diets containing MOL may be related to the higher amount of this type of fiber in the diets. NSP, with their adverse effects on digestion and intestinal transit, may have caused this portion of the gastrointestinal tract to enhance its secretory mechanisms, due to the increase in the amount of undigested substrate and, consequently, a greater need for digestive enzymes, which triggered an increase in total intestinal size and the weight of attached glands (23, 25). The increase in viscosity caused by soluble NSP stimulates the secretion of pancreatic juice and increases the spacing of the intestinal mucosal barrier, which hinders the contact of enzymes with substrates, thus impairing the formation of micelles and the digestion of lipids, fats, and carbohydrates (54). According to Sousa et al. (33), soluble NSP increase the metabolic activity of the liver, resulting in enhanced synthesis and secretion of bile acids due to the affinity of this type of fiber for these compounds. This increases their excretion and causes greater liver activity to restore normal levels of bile acids in the gastrointestinal tract, which can lead to increases in liver size.

When xylanase was added to the feed, the NSP were degraded, reducing the relative size of the glands, small intestine, and caeca. Other studies have shown a reduction in the size of the liver and pancreas when xylanase is supplemented in diets containing fibrous poultry feed (23, 55). Hoseini et al. (56) observed shorter small intestine lengths in birds fed wheat-based diets supplemented with carbohydrases. In this study, there were no effects of phytase on decreasing the weight or length of the intestines and glands, indicating that the observed results were mainly due to supplementation with xylanase, either individually or in combination.

The gizzard is an essential organ in feed digestion in poultry; it has strong and thick muscles with the primary function of mechanical digestion for grinding and, consequently, reducing the particle size of the food (20). *Moringa oleifera* leaves can modify the anatomical structure of the birds’ gastrointestinal tract due to their fibrous content (57, 58). It has already been reported that birds need a minimum amount of fiber to maintain the function of the gizzard and the activity of the gastrointestinal tract (59), which should not exceed levels greater than 5%, according to the recommendations in the manual for the strain used in this study. The inclusion of fiber in the diet improves the development of the gizzard, as the presence of these components in this segment increases its growth, enhances the motility of the digestive tract, and increases the secretion of cholecystokinin (CCK), thus improving the mixing of digestive enzymes with the digesta (10, 22, 60–62). The increase in fiber levels present in the rations containing MOL (Table 2) and the increased activity of the proventriculus to grind these components provide a plausible explanation for the increase in gizzard weight when the leaves were included in the diet. Teteh et al. (57) observed significant increases in the weight of the proventriculus at 56 days of age when they fed laying hens with increasing levels of MOL. However, the authors of that study did not specify the amount of fiber present in the leaves used; the determined levels of crude fiber in the laying rations did not exceed 5%. When xylanase was added to the feed, it degraded NSPs, reducing the relative size of the glands, small intestine, and caeca. Similar reductions in liver and pancreas size with xylanase supplementation in fibrous diets have been reported (23, 55). Hoseini et al. (56) also observed shorter small intestines in birds fed wheat-based diets with carbohydrases. In contrast, phytase had no significant effect on the weight or length of intestines and glands in this study, indicating that the changes were mainly due to xylanase supplementation, alone or combined.

The primary function of the gastrointestinal tract is to promote the digestion and absorption of nutrients for maintenance, growth, and production. Maintaining intestinal health is crucial for profitable and sustainable production systems, as disorders can negatively impact production efficiency, animal welfare, and environmental protection (63). The duodenum, jejunum, and ileum are key sites of digestion and nutrient absorption, with their surface area and epithelial properties influencing absorption capacity (30, 64, 65). Morphological characteristics such as villus height (VH), crypt depth (C), villus height/crypt depth ratio (V:C), mucosal length (ML), and villus width (VW) are commonly used to assess intestinal functional capacity and response to diet.

NSPs adversely affect the enteric mucosa, causing villus shortening and widening, reduced crypt depth, mucosal atrophy, and increased goblet cells, all impairing intestinal function (26, 28). Consistent with this, the present study found that NSPs in *Moringa oleifera* leaf meal (MOL) negatively influenced the small intestine histomorphology, evidenced by significant reductions in VH, C, V:C, ML, and VW across the duodenum, jejunum, and ileum of laying hens.

In contrast, supplementation with xylanase—either alone or in combination with phytase—mitigated these negative effects and significantly improved all evaluated intestinal parameters. These improvements align with previous findings reporting that xylanase enhances intestinal morphology by hydrolyzing NSPs and reducing their antinutritional effects (56, 66, 67). For example, the increased VH observed in our study suggests a greater number of enterocytes and enteroendocrine cells, along with enhanced expression of brush border enzymes, which contribute to improved digestion and nutrient absorption (68, 69).

Furthermore, our findings support the interpretation by Yason et al. (70), who stated that crypt depth reflects the regenerative activity of the intestinal lining. In our study, the greater crypt depth observed with xylanase supplementation indicates a healthier mucosal renewal process compared to the MOL-only treatment, where crypt depth was significantly reduced. Likewise, the lower VH and crypt depth seen in the MOL treatment are indicative of impaired absorption, while the higher V:C ratio in the xylanase-supplemented groups points to a more developed and functionally efficient intestinal mucosa (71). Similarly, the increases in villus width observed with xylanase supplementation in our study are consistent with literature linking wider villi to enhanced nutrient absorption due to increased surface area (30, 72).

Although the NSP content of the diets was not directly measured in this study, the significant improvements in intestinal morphology following xylanase supplementation strongly suggest the presence of hydrolysable NSP substrates in the MOL-based diets. This further supports findings from previous studies demonstrating the beneficial effects of xylanase in improving intestinal morphometry in poultry fed fibrous diets (23, 30, 65, 72–74). Thus, our results confirm that xylanase plays an important role in attenuating the negative effects of dietary fiber and in promoting intestinal health in laying hens fed MOL-containing diets.

However, it seems that MOL, depending on the supplementation level, maturity stage, and gastrointestinal tract section of the birds, exert a positive influence on the morphological characteristics of the intestines of these animals (35). These same researchers observed an increase in VH and the V:C ratio of the jejunum and ileum of broiler chickens fed diets containing 1.2% MOL, with a decline at higher inclusion levels. It should be noted that laying hens, being older animals and consequently having a more mature gastrointestinal tract, have a greater capacity to digest fiber. The effects observed in this study, where MOL without enzyme supplementation was able to increase VH, C, and ML of the jejunum, as well as ML and the V:C ratio of the ileum, show that the birds, even when fed diets without enzyme supplementation, seem to tolerate higher levels of fiber without significant impairment of the digestive and absorptive processes. In addition, *Moringa oleifera* leaves contain L-glutamine, an amino acid derivative of glutamate, which plays an important role in maintaining the integrity of the intestinal mucosa (75, 76). According to Rao and Sama (76), L-glutamine increases the rate of protein synthesis in the intestine, reduces proteolysis in enterocytes, and is used as an energy source for the proliferation of intestinal epithelial cells, which promotes greater intestinal integrity and, consequently, an improvement in intestinal morphology. Other studies have found better morphological characteristics in all sections of the intestines of birds fed MOL at inclusion levels ranging from 1.0 to 5.0% (36, 75, 77–80).

The effects of phytase on intestinal morphology observed in this study were limited and primarily restricted to the jejunum, where the enzyme increased villus height (VH), crypt depth (C), and mucosal length (ML) in diets containing *Moringa oleifera* leaf meal (MOL). Phytate levels in *Moringa* leaves can reach approximately 2.5% (81), and this compound is known to form insoluble complexes with minerals such as phosphorus, calcium, magnesium, iron, and zinc, as well as with proteins and other nutrients (82–85). Phytase supplementation has been reported to improve intestinal morphology mainly by reducing the anti-nutritional effects of phytate. This occurs through the hydrolysis of phytate, which not only enhances nutrient bioavailability but also limits the amount of substrate available for pathogenic microbial fermentation. Consequently, phytase may help modulate gut microbiota composition, reduce the inflammatory response, and minimize mucosal damage, thereby contributing to improved epithelial structure and function (86–88). In this study, such effects were evident through localized improvements in the jejunal mucosa of laying hens fed MOL-based diets.

## 5 Conclusion

MOL inclusion in the diet had multiple effects on the GIT of laying hens, including increasing the size of intestinal segments and associated glands, as well as modulating key histomorphological parameters. The supplementation of xylanase, through its ability to degrade NSP, contributes to the restoration of intestinal integrity and function, potentially improving nutrient absorption and overall performance in laying hens. However, further studies are warranted to characterize the specific NSP profile of *Moringa oleifera* leaves to better understand their interactions with exogenous enzymes and optimize their inclusion in poultry diets.

## Data Availability

The original contributions presented in the study are included in the article/supplementary material, further inquiries can be directed to the corresponding author.
